# Supplementary material for: The ‘Double Helix’ model of quality monitoring: Risk mapping of quality management system during initial ISO 15189 Implementation in a medical laboratory
Source: PLoS One. 2026 Feb 4;21(2):e0342129. doi: 10.1371/journal.pone.0342129 (PMC12871953; doi:10.1371/journal.pone.0342129)
Supplement: S1 Table — (DOC) [file pone.0342129.s001.doc]

| Sub-dimensions | Internal assessment(N) | | | | | ***External assessment(N)***** | | | |
| --- | --- | --- | --- | --- | --- | --- | --- | --- | --- |
|  | 2021 | 2022 | 2023 | 2024 | Total(N/%) | ***2022*** | ***2023*** | ***2024*** | ***Total(N/%)*** |
| Documents and records | 3 | 3 | 0 | 0 | 6/17.14 | ***6*** | ***2*** | ***2*** | ***10/14.93*** |
| Personnel | 2 | 1 | 2 | 2 | 7/20.00 | ***2*** | ***2*** | ***2*** | ***6/8.96*** |
| Facilities and environmental conditions | 0 | 0 | 0 | 0 | 0/0 | ***1*** | ***1*** | ***2*** | ***4/5.97*** |
| Equipment | 4 | 1 | 0 | 0 | 5/14.28 | ***3*** | ***1*** | ***1*** | ***5/7.46*** |
| Reagent and consumables | 2 | 1 | 1 | 1 | 4/11.43 | ***3*** | ***3*** | ***0*** | ***6/8.96*** |
| Pre-examination processes | 0 | 1 | 0 | 1 | 2/5.71 | ***2*** | ***1*** | ***0*** | ***3/4.48*** |
| Examination processes | 2 | 3 | 0 | 2 | 8/22.86 | ***6*** | ***3*** | ***10*** | ***19/28.36*** |
| Post-examination processes | 0 | 0 | 0 | 1 | 1/2.86 | ***3*** | ***3*** | ***0*** | ***6/8.96*** |
| Others* | 1 | 1 | 0 | 0 | 2/5.71 | ***3*** | ***2*** | ***3*** | ***8/11.94*** |
| Total | 14 | 11 | 3 | 7 | 35/100 | ***29*** | ***18*** | ***20*** | ***67/100*** |

*Others: Evaluation,Complaints,Information system, Risk Management

****Data in bold italics are from external assessments.**
