# Supplementary material for: The ‘Double Helix’ model of quality monitoring: Risk mapping of quality management system during initial ISO 15189 Implementation in a medical laboratory
Source: PLoS One. 2026 Feb 4;21(2):e0342129. doi: 10.1371/journal.pone.0342129 (PMC12871953; doi:10.1371/journal.pone.0342129)
Supplement: S2 Table — (DOC) [file pone.0342129.s002.doc]

| Sub-dimensions | Internal assessment(N) | | | | | ***External Vassessment(N)****  ***Total(N/%)*** | | | |
| --- | --- | --- | --- | --- | --- | --- | --- | --- | --- |
|  | 2021 | 2022 | 2023 | 2024 | Total(N/%) | ***2022*** | ***2023*** | ***2024*** | ***Total(N/%)*** |
| Document and Record Deficiencies | 1 | 0 | 1 | 0 | 2/5.71 | ***6*** | ***4*** | ***5*** | ***15/22.39*** |
| Training Deficiencies | 6 | 4 | 0 | 1 | 11/31.43 | ***13*** | ***9*** | ***5*** | ***27/40.30*** |
| Process Deficiencies | 2 | 0 | 0 | 3 | 5/14.29 | ***3*** | ***2*** | ***2*** | ***7/10.45*** |
| Personnel Negligence | 4 | 6 | 2 | 2 | 14/40.00 | ***6*** | ***1*** | ***7*** | ***14/20.90*** |
| Resource Constraints | 1 | 1 | 0 | 1 | 3/8.57 | ***1*** | ***2*** | ***1*** | ***4/5.97*** |
| Total | 14 | 11 | 3 | 7 | 35/100 | ***29*** | ***18*** | ***20*** | ***67/100*** |

***Data in bold italics are from external assessment.**
