# Supplementary material for: The ‘Double Helix’ model of quality monitoring: Risk mapping of quality management system during initial ISO 15189 Implementation in a medical laboratory
Source: PLoS One. 2026 Feb 4;21(2):e0342129. doi: 10.1371/journal.pone.0342129 (PMC12871953; doi:10.1371/journal.pone.0342129)
Supplement: S3 Table — (DOC) [file pone.0342129.s003.doc]

| Sub-dimensions | Internal assessment(N) | | | | | ***External assessment(N)**** | | | |
| --- | --- | --- | --- | --- | --- | --- | --- | --- | --- |
|  | 2021 | 2022 | 2023 | 2024 | Total(N/%) | ***2022*** | ***2023*** | ***2024*** | ***Total(N/%)*** |
| Personnel training | 14 | 11 | 3 | 7 | 35/47.29 | ***29*** | ***18*** | ***20*** | ***67/41.36*** |
| Document modification | 1 | 0 | 1 | 0 | 2/2.70 | ***8*** | ***7*** | ***8*** | ***23/14.20*** |
| Supervision and inspection | 4 | 0 | 0 | 2 | 6/8.12 | ***5*** | ***4*** | ***4*** | ***13/8.02*** |
| Assessing the impact of NCs | 0 | 0 | 0 | 0 | 0/0 | ***3*** | ***3*** | ***1*** | ***7/4.32*** |
| LIS modification | 0 | 1 | 0 | 0 | 1/1.35 | ***4*** | ***2*** | ***1*** | ***7/4.32*** |
| Supplementary records | 10 | 10 | 3 | 5 | 28/37.84 | ***0*** | ***2*** | ***0*** | ***2/1.23*** |
| Total | 29 | 22 | 7 | 14 | 72/97.30 | ***49*** | ***36*** | ***34*** | ***119/73.46*** |

***Data in bold italics are from external assessments.**
