# Supplementary material for: The ‘Double Helix’ model of quality monitoring: Risk mapping of quality management system during initial ISO 15189 Implementation in a medical laboratory
Source: PLoS One. 2026 Feb 4;21(2):e0342129. doi: 10.1371/journal.pone.0342129 (PMC12871953; doi:10.1371/journal.pone.0342129)
Supplement: Attachment — Submitted filename: Reviewer report-.docx [file pone.0342129.s004.docx]

**Reviewer report:**

**The 'Double Helix' Model of Quality Monitoring:Risk Mapping of quality management system During Initial ISO 15189 Implementation in a medical laboratory**

The study provides a valuable, data-driven exploration of quality monitoring during the early stages of ISO 15189 implementation. The authors successfully highlight high-risk areas, the role of assessments, and the importance of training and documentation. However, single-laboratory-scope, internal bias risks, and limited recurrence analysis suggest areas for improvements in future research.

The study include many strength outcomes. The objective is clear and relevant as the study addresses a critical phase in laboratory quality management, the initial implementation of ISO 15189, which is often unstable and prone to systemic vulnerabilities. The study aligns with global efforts to strengthen laboratory services, especially in the context of evidence-based medicine and industry 4.0. The study shed the light on the internal and external assessments and compare between them, providing valuable insights into the efficacy, depth and scope of each method. Furthermore, the use of statistical tests (Mann-Whitney U, fisher’s exact test) adds rigor to the comparative analysis. Using Sankey diagrams and trend charts effectively visualize relationships and temporal patterns among quantitative data from 102 nonconformities (102). The identification of training deficiencies and documentation issues as major root causes is consistent with known challenges in QMS implementation. The study also evaluates the effectiveness of corrective actions, which is crucial for continues improvement. The discussion integrates national challenges in China, offering insights for similar settings. However, the study includes some limitations which are worthy to be mentioned here.

- The study is based on data from one medical laboratory, limiting generalizability. Multicentre validations needed to confirm findings across diverse settings.
- Internal assessors may have conflicts of interest or blind spots, especially in areas like documentations where they are also authors. The training duration and rigor for internal assessors were notably less than for external assessors, possibly affecting assessment quality.
- Limited exploration of recurrence causes as the rates were analysed, the qualitative exploration of why corrective actions failed, for instance poor root cause analysis, could be further expanded.
- The authors misidentified the root causes but failed to propose a structured framework to improve it.
- Smal sample size in certain categories could be the reason for insignificant statistical rates among some comparison, such as recurrence rates, effectiveness rates.
- Could the authors suggest or develop digital tools or automation for the ISO updates to sustainability.
- The title is engaging “Double Helix Model” but lacks clarity on what the model entails.
- In the method sections, the retrospective design is appropriate, but the rationale for selecting the specific time frame (2021-2024) and the number of assessments (4 internals, 3 externals) should be clarified.
- The use of Sankey diagrams is innovative for visualizing flow relationships. However, the methodology for constructing these diagrams is not described in detail.
- The terms “recurrence rate and corrective action effectiveness” are provided, but the formulas could be more clearly formatted for readability.
- In the result sections, table 1 is dense and could benefit from clearer subheadings or colour coding to distinguish internal VS. external data.
- Effect size or confidence intervals are missing, which limit interpretation of practical relevance.
- Table 4 is insightful but underexplored. More discussion on why certain NCs recur despite corrective actions strengthen the findings.
- Although, authors acknowledge its single-centre limitation, which is commendable. However, it could propose specific strategies for multicentre validation.
- The ethical approval or data protection is missing, which are important even in retrospective analyses.
- Checklist templates and training modules are needed in addition to the practical recommendation.
