# Supplementary material for: The ‘Double Helix’ model of quality monitoring: Risk mapping of quality management system during initial ISO 15189 Implementation in a medical laboratory
Source: PLoS One. 2026 Feb 4;21(2):e0342129. doi: 10.1371/journal.pone.0342129 (PMC12871953; doi:10.1371/journal.pone.0342129)
Supplement: Attachment — Submitted filename: Reviewer PLOS ONE1.docx [file pone.0342129.s005.docx]

### ****General Overview****

This manuscript presents a comparative analysis of nonconformities (NCs), root causes, and corrective actions identified during internal and external assessments in a medical laboratory implementing ISO 15189 for the first time. The authors introduce a conceptual “Double Helix” model that represents the complementary relationship between internal and external quality assessments.

The topic is highly relevant to laboratory accreditation and risk-based quality management, especially given the 2022 revision of ISO 15189. The study is well-structured and supported by quantitative analysis, but it requires **major language revision and moderate methodological clarification** before publication.

## ****Comments to the Editor****

The manuscript is scientifically relevant and contributes to ongoing discussions about risk-based laboratory quality management under ISO 15189:2022. While the core data are strong, the current presentation and language issues hinder accessibility. I recommend inviting a **major revision** focused on language quality, conceptual clarity (particularly the “Double Helix” model), and data presentation improvements.

**Comments to the Author**

1. **Clarity and Language**

There are a lot of typographical, grammatical, and formatting mistakes in the document (such as "the datas," "the potential," and "supervision/inspectionin"). Sometimes sentences are lengthy and complicated, making them harder to read. To improve clarity and flow, please have a professional scientific editor or native English speaker perform thorough English language editing.

2. **"Double Helix" Conceptual Model**

Although the "Double Helix" concept is an intriguing and inventive parallel, it is not well defined. Describe the model's theoretical underpinnings and how internal and external assessments work together as "strands" of ongoing quality improvement. Provide a schematic picture of the model to illustrate its functioning within the framework of ISO 15189 implementation.

3. **Methodological Details**

Make clear if inclusion/exclusion criteria were used or if all nonconformities (NCs) found between 2021 and 2024 were examined. Give a more thorough explanation and rationale, preferably with references, for the formulas used to determine the Recurrence Rate and Corrective Action Effectiveness Rate. Describe the methods used to assure data reliability, such as independent reviewers and double-checking NC classification. Talk about the limits that come with researching a specific institution and how this could impact generalizability.

4. **Data Presentation and Statistics**

a. When applicable, assumptions, sample distributions, and effect sizes should be included in the explanation of the statistical tests (Mann-Whitney U and Fisher's exact test).
b. Reformat tables to improve readability and uniformity; some cells have uneven alignments and combine text and numerical data.

c. Figures, particularly Sankey diagrams, ought to include stand-alone captions that completely explain the contents and more lucid legends.

5. **Discussion and Interpretation**

a. The practical implications of the findings for laboratories at various degrees of QMS development should be further explored.

b. Strengthen the connection between the findings and the body of research on ISO 15189 implementation and QMS evolution in various resource or geographic situations.
c. Extend the limits section to include information about the findings' contextual specificity, limited dataset, and possible assessor bias.
