# Supplementary material for: The ‘Double Helix’ model of quality monitoring: Risk mapping of quality management system during initial ISO 15189 Implementation in a medical laboratory
Source: PLoS One. 2026 Feb 4;21(2):e0342129. doi: 10.1371/journal.pone.0342129 (PMC12871953; doi:10.1371/journal.pone.0342129)
Supplement: Attachment — Submitted filename: Response to Reviewers.doc [file pone.0342129.s006.doc]

Dear Editor:

Thank you very much for taking the time to review our manuscript and for providing us with such valuable and constructive comments. In revising our article according to your suggestions, our team has not only gained a deeper understanding of quality management systems in medical laboratories but also developed more rigorous scientific thinking. This process has provided us with more practical directions for our future research.

If some revisions still fall short of your expectations, possibly due to our occasional misinterpretation of certain points. We sincerely aimed to reflect the growth and insights our team has gained throughout this revision process in the revised manuscript. If the revision progress has been slower than expected, we kindly ask for your understanding.

Once again, we extend our heartfelt gratitude to you and the reviewers for your careful guidance. It is with your support that the academic depth and practical value of this study have been significantly enhanced.We hope you had a wonderful holiday season and wish you all the best for the coming New Year.

**Journal Requirements:**

Please ensure that your manuscript meets PLOS ONE's style requirements, including those for file naming. The PLOS ONE style templates can be found at https://journals.plos.org/plosone/s/file?id=wjVg/PLOSOne_formatting_sample_main_body.pdf and  <https://journals.plos.org/plosone/s/file?id=ba62/PLOSOne_formatting_sample_title_authors_affiliations.pdf>

We ensure our manuscript meets PLOS ONE's style requirements.

2. We note that your Data Availability Statement is currently as follows:

“All relevant data are within the manuscript and its Supporting Information files.”

Please confirm at this time whether or not your submission contains all raw data required to replicate the results of your study. Authors must share the “minimal data set” for their submission. PLOS defines the minimal data set to consist of the data required to replicate all study findings reported in the article, as well as related metadata and methods (https://journals.plos.org/plosone/s/data-availability#loc-minimal-data-set-definition).

For example, authors should submit the following data:

- The values behind the means, standard deviations and other measures reported;
- The values used to build graphs;
- The points extracted from images for analysis.

Authors do not need to submit their entire data set if only a portion of the data was used in the reported study.

If your submission does not contain these data, please either upload them as Supporting Information files or deposit them to a stable, public repository and provide us with the relevant URLs, DOIs, or accession numbers. For a list of recommended repositories, please see https://journals.plos.org/plosone/s/recommended-repositories.

If there are ethical or legal restrictions on sharing a de-identified data set, please explain them in detail (e.g., data contain potentially sensitive information, data are owned by a third-party organization, etc.) and who has imposed them (e.g., an ethics committee). Please also provide contact information for a data access committee, ethics committee, or other institutional body to which data requests may be sent. If data are owned by a third party, please indicate how others may request data access.

We are uploading the raw data from this study to the specified website/platform.

3. PLOS requires an ORCID iD for the corresponding author in Editorial Manager on papers submitted after December 6th, 2016. Please ensure that you have an ORCID iD and that it is validated in Editorial Manager. To do this, go to ‘Update my Information’ (in the upper left-hand corner of the main menu), and click on the Fetch/Validate link next to the ORCID field. This will take you to the ORCID site and allow you to create a new iD or authenticate a pre-existing iD in Editorial Manager.

We have the ORCID iD .

4.Please ensure that you refer to Figures 1 and 2 in your text as, if accepted, production will need this reference to link the reader to the figure.

Thank you for the reminder. We have ensured that Figures 1 and 2 are now appropriately cited within the main text of the revised manuscript.

5. If the reviewer comments include a recommendation to cite specific previously published works, please review and evaluate these publications to determine whether they are relevant and should be cited. There is no requirement to cite these works unless the editor has indicated otherwise.

Thank you for this note.The reviewers did not recommend any specific publications for citation but good suggestions.

**Reviewer #2: 1. Clarity and Language**
1.There are a lot of typographical, grammatical, and formatting mistakes in the document (such as "the datas," "the potential," and "supervision/inspectionin"). Sometimes sentences are lengthy and complicated, making them harder to read. To improve clarity and flow, please have a professional scientific editor or native English speaker perform thorough English language editing.

We sincerely thank the Editors for their meticulous and valuable comments regarding the language quality of our manuscript.We have proofread and corrected typographical, grammatical, and formatting errors in the document.We have restructured all lengthy and complex sentences, breaking them into shorter sentences.We apologize for the deficiencies in the initial submission and are deeply grateful for your guidance and assistance throughout this process.

2. "Double Helix" Conceptual Model
   Although the "Double Helix" concept is an intriguing and inventive parallel, it is not well defined. Describe the model's theoretical underpinnings and how internal and external assessments work together as "strands" of ongoing quality improvement. Provide a schematic picture of the model to illustrate its functioning within the framework of ISO 15189 implementation.

We agree that the “Double Helix” concept, as an innovative analogy, required clearer definition and elaboration. In this revision, we have substantively addressed these points by grounding the model ***Fig.4 in Result section (Page9)*** and elaborating its function in ***Discussion section (Page12)***.1.Providing a Schematic Based on Results : Based on our analysis of the characteristics and interrelationships among NCs, root causes, and corrective actions from both internal and external assessments, we have introduced a schematic of the “Double-Helix Model” ***in the last Result section(Page9) .*** This Fig. 4 visually depicts how the internal and external assessment interlinked.2. Deepening Theoretical Explanation of the Model : we have added ***paragraph 7 In the Discussion section (Page12)*** to systematically explain the model’s core mechanism. We discuss how internal and external assessments function as complementary “strands.This collaborative interaction provides dynamic stability and a continuous upward driving force for the quality management system.

We are grateful for your guidance, which has been instrumental in enhancing the completeness and scholarly contribution of this work.

3.Methodological Details
Make clear if inclusion/exclusion criteria were used or if all nonconformities (NCs) found between 2021 and 2024 were examined. Give a more thorough explanation and rationale, preferably with references, for the formulas used to determine the Recurrence Rate and Corrective Action Effectiveness Rate. Describe the methods used to assure data reliability, such as independent reviewers and double-checking NC classification. Talk about the limits that come with researching a specific institution and how this could impact generalizability.

We have carefully considered all comments and have substantially revised the methodology section to enhance clarity, rigor, and reproducibility. The key modifications in the revised manuscript are detailed below.We have revised the "Method" section (page2,3)to provide explicit clarification. The "Method" section has been restructured into four distinct parts for enhanced clarity.Data collection***(Page2)***: we explicitly state that the use of data was approved by the Deputy Director of the laboratory. Furthermore, it is clarified that all nonconformities (NCs) identified and recorded between 2021 and 2024 were comprehensively examined for this analysis.We have provided detailed explanations for the classification of NC types, root causes, and corrective actions. A dual-review process was employed to ensure data reliability.Data Analysis***(page3):*** We have elaborated on the calculation methods for the two rates and provided detailed definitions for all concepts involved in the formulas.

We fully acknowledge that as a single-center study, the generalizability of our findings is subject to certain limitations. We have addressed content ***in the last*** ***Paragraph of the Discussion section (Page 12)***, which primarily includes the following aspects:1.Constraints of Institution-Specific Factors.2.Variability in Standard Implementation and Auditing, auditor competency and judgment.3.Inherent Limitations of the Methodology.we have suggested directions for future research in the manuscript, such as extending the observation period, quantifying changes in fundamental laboratory conditions and incorporating them as covariates in the analysis.To address these limitations and enhance the utility of our work, we explicitly recommend:When other laboratories apply the indicator framework and analytical methods developed in this study, they should first conduct a systematic evaluation of their own specific conditions and make necessary local adaptations. We wish that openly acknowledging these limitations and their implications does not diminish the value of our study ,but, rather, helps to clearly define its scope of application and provides a clear pathway for subsequent research. Thank you again for helping us enhance the academic rigor of this work.

4.Data Presentation and Statistics
a. When applicable, assumptions, sample distributions, and effect sizes should be included in the explanation of the statistical tests (Mann-Whitney U and Fisher's exact test).
b. Reformat tables to improve readability and uniformity; some cells have uneven alignments and combine text and numerical data.
c. Figures, particularly Sankey diagrams, ought to include stand-alone captions that completely explain the contents and more lucid legends.

a.We have carefully addressed the reviewer’s point regarding the explanation of statistical tests. Specifically, we have revised the ‘***Statistical Methods***’ subsection in the ‘Methods’ section ***(Page3)***to provide a comprehensive explanation of the Mann-Whitney U test and Fisher’s exact test.The revisions explicitly include the following for each test:1.Assumptions: We state the core assumptions (e.g., independence of observations for both tests; the use of Fisher’s test when expected cell counts are <5).2.Sample Distributions: We clarify the conditions of our data that guided the test selection (e.g., use of non-parametric tests due to non-normally distributed count data and small sample sizes).3.Effect Sizes: We specify the effect size measures reported for significant results (the rank-biserial correlation coefficient for the Mann-Whitney U test and the odds ratio with its 95% confidence interval for Fisher’s exact test).These clarifications significantly enhance the methodological rigor and transparency of our study.

b.We have comprehensively revised all tables in the manuscript: text is left-aligned, and numerical data are center-aligned.We have split ***Table 1 (Page4)*** into three separate tables to present the content more clearly. Additionally, we have formatted the external assessment data in bold italics to facilitate differentiation.For the last table(Page9), we retained the classification of NCs to clearly illustrate the nature of recurring NCs. If you feel this approach is not suitable, we are very willing to further revise this table to achieve greater clarity and accuracy.

We have divided the Sankey figures into two sequential sections ***(Page7-8)***. Each set of diagrams includes separate charts for internal and external assessments.We have provided captions for both Fig. 2 and Fig. 3***(Page8) .***These captions clearly define the variables represented in each column, highlight the most frequent data flows and their counts, and include a brief comparative analysis between internal and external assessments.We wish these revisions significantly improve the clarity and professionalism of the figures.

5. Discussion and Interpretation
a. The practical implications of the findings for laboratories at various degrees of QMS development should be further explored.
b. Strengthen the connection between the findings and the body of research on ISO 15189 implementation and QMS evolution in various resource or geographic situations.
c. Extend the limits section to include information about the findings' contextual specificity, limited dataset, and possible assessor bias.

a.Thank you for this valuable suggestion. We have added the content to ***Paragraph7 of the Discussion section (Page 12).***Differential Implications for Initial Stage Laboratories: For laboratories in the initial establishment phase, our results highlight the core guiding role of external assessments in identifying systemic deviations and establishing a compliance foundation, as well as the critical value of internal audits in horizontally extending supervision and consolidating improvements.

b.We have specifically cited evidence from studies in settings such as Hongkong (***Paragraph2 of the Discussion section (Page 10)***)，Kenya and Indiain***（Paragraph8 of the Discussion section (Page 12))*** .We wish these additions significantly deepen the practical value of our research, enabling its conclusions to provide more targeted guidance for laboratories with different levels of development and resource backgrounds.

c.To more comprehensively delineate the limitation of our study, we have made substantial expansions to ***Paragraph10 of the Discussion section (Page 12).***

6. PLOS authors have the option to publish the peer review history of their article ([what does this mean?](https://journals.plos.org/plosone/s/editorial-and-peer-review-process#loc-peer-review-history)). If published, this will include your full peer review and any attached files.

We choose “YES”

Reviewer #1:

The study is based on data from one medical laboratory, limiting generalizability. Multicentre validations needed to confirm findings across diverse settings.

To more comprehensively delineate the limitation of our study, we have made substantial expansions to ***Paragraph9 of the Discussion section (Page 12).***

Internal assessors may have conflicts of interest or blind spots, especially in areas like documentations where they are also authors.

Thank you for raising this important and insightful point. We fully agree that internal assessors, particularly when they are also the authors of the documents under review, face potential conflicts of interest and blind spots in the evaluation of documentation and records. This is an inherent challenge of internal auditing.

We have explicitly addressed this issue in ***Paragraph3 of the Discussion section (Page10-11)*** To effectively mitigate this concern, we have proposed a comprehensive operational plan comprising four core measures:Strictly Enforce an "Author Recusal" and "Cross-Review" System

We wish this comprehensive strategic framework can effectively identify, manage, and mitigate the potential bias and blind spots of internal assessors in document review.

The training duration and rigor for internal assessors were notably less than for external assessors, possibly affecting assessment quality.

Thank you for raising this insightful point. We have incorporated an analysis of the assessors‘ potential influence into the revised discussion section.In response to this issue, we have made limitations to  ***Paragraph9 of the Discussion section (Page 12)***. We explicitly state that differences may exist between internal and external assessors regarding their depth of understanding of ISO 15189 clauses and their tolerance thresholds for deviations in the quality system.We wish this addition not only directly addresses your specific concern but also enhances the methodological rigor and transparency of our study's self-reflection. We thank you for helping us strengthen this important discussion.

Limited exploration of recurrence causes as the rates were analysed, the qualitative exploration of why corrective actions failed, for instance poor root cause analysis, could be further expanded.

In the ***Paragraph 5 of the Discussion section(Page 11-12)***, we have substantially expanded and rewritten the analysis regarding the failure of corrective actions and the root causes of NC recurrence. Based on a meticulous review of the data and team discussion, the new analysis primarily reveals the following four key systemic causes and proposes corresponding improvement strategies:

1.Insufficient Scope of Corrective Action Implementation: Corrective actions were often confined to the specific professional group where the NC was initially identified (e.g., the "Reagents and Consumables" group). Due to the limitations of audit sampling or variations in assessor focus, similar issues in other groups were not systematically investigated.2.

Superficial and Biased Review of Similar Issues: Even when reviews were extended to other groups, the approach was often superficial, and there may have been a tendency to select well-performing groups or assays to expedite NC closure (e.g., for internal quality control failures in "Examination Processes").3.Inadequate Verification of Training Effectiveness: Recurrence rates were highest for NCs related to "Personnel Negligence" and "Training Deficiencies." We recommend face-to-face, repeated training delivered by trainers with strong system knowledge, and conducting assessments on a separate day from the training.4.Cross-Departmental Coordination Gaps: Some corrective actions intersected with other hospital departments but were implemented without adequately considering their workflows (e.g., equipment management), hindering effective implementation.

We wish these additions significantly deepen the understanding of why NCs recur. We thank you again for your guidance, which has been instrumental in greatly enhancing the depth and value of this analysis.

The authors misidentified the root causes but failed to propose a structured framework to improve it.

We have added a comprehensive operational plan specifically designed to effectively mitigate root causes in the ***Paragraph3 of the Discussion section (Page10-11)***. This plan constitutes a clear, structured improvement framework.

1.A Systematic Training Optimization Framework for “Personnel Negligence and Training Deficiencies”:
Addressing the high recurrence linked to inadequate training effectiveness, we have further proposed a systematic training optimization framework. This framework is built around four actionable pillars: (1) Content Comprehensiveness, (2) Trainer Competency, (3) Format Effectiveness, and (4) Assessment Validity. We specifically emphasize that training needs to be repeated, with increased frequency for high-risk areas, and recommend conducting assessments on a separate day from training to ensure their validity.

 2.A Structured Operational Plan for “Document and Record Deficiencies”::1.Strictly enforce an “author recusal” and “cross-review” system2.Conduct periodic, systematic document and record review campaigns。3.Regularly engage external experts to perform evaluations.4.Establish a fully traceable lifecycle management system for documents, providing technological support.

We wish this structured framework not only directly responds to your concern but also provides laboratory managers with concrete, actionable guidance.

Smal sample size in certain categories could be the reason for insignificant statistical rates among some comparison, such as recurrence rates, effectiveness rates.

In response to your comment, we have added the content to the “Limitations” in the ***Paragraph9 of the Discussion section (Page 12)***. Added text: “Certain subgroup analyses may have been underpowered due to low event counts. This could have prevented the detection of statistically significant differences. Therefore, interpretations of these subgroup comparisons based on small samples should be made with caution.”

We wish that by explicitly acknowledging this limitation and guiding readers to interpret the relevant results with caution, our analysis gains in rigor and transparency. We thank you again for helping us improve the methodological presentation of our paper.

Could the authors suggest or develop digital tools or automation for the ISO updates to sustainability.

we have added the content to the ***last Paragraph of the Discussion section (Page 12)***. The new content explicitly proposes developing an AI-powered automated quality assessment system as a core technological pathway to address standard updates and enhance management efficiency. Specifically, it includes:1.Leveraging Artificial Intelligence to Screen Sensitive Quality Indicators: Utilizing AI algorithms (e.g., machine learning) to automatically identify key indicators most sensitive to new requirements, such as sustainability, from vast operational data, enabling precise and early warning of deviations in the quality system.2.Establishing an Automated, Intelligent Quality Assessment System: Developing an integrated, AI-enabled automated system. This system would be capable of conducting impartial, comprehensive, and consistent evaluations of laboratory quality performance against the latest standard requirements (including sustainability clauses). It would reduce the subjectivity and lag inherent in manual audits and enable automated monitoring of compliance with new requirements.

We are grateful for your suggestion, which has significantly enhanced the practical foresight of our research.

The title is engaging “Double Helix Model” but lacks clarity on what the model entails

We agree that the “Double Helix” concept, as an innovative analogy, required clearer definition and elaboration. In this revision, we have substantively addressed these points by grounding the model ***Fig.4 in Result section (Page9)*** and elaborating its function in ***Discussion section (Page12)***.1.Providing a Schematic Based on Results : Based on our analysis of the characteristics and interrelationships among NCs, root causes, and corrective actions from both internal and external assessments, we have introduced a schematic of the “Double-Helix Model” ***in the last Result section(Page9) .*** This Fig. 4 visually depicts how the internal and external assessment interlinked.2. Deepening Theoretical Explanation of the Model : we have added ***paragraph 7 In the Discussion section (Page12)*** to systematically explain the model’s core mechanism. We discuss how internal and external assessments function as complementary “strands.This collaborative interaction provides dynamic stability and a continuous upward driving force for the quality management system.

We are grateful for your guidance, which has been instrumental in enhancing the completeness and scholarly contribution of this work.

In the method sections, the retrospective design is appropriate, but the rationale for selecting the specific time frame (2021-2024) and the number of assessments (4 internals, 3 externals) should be clarified.

Thank you for raising this important point. we have added a new subsection titled ***“Laboratory Settings” in the Methods section (page2)*** to explicitly explain the reasons for selecting the 2021–2024 time frame .

The key rationales are as follows:

Rationale for the Time Frame (2021–2024): This period was deliberately chosen to completely span the critical transition phase of our laboratory’s QMS: from the “initial establishment phase” (initiation of formal, ISO 15189-aligned internal audits in 2021), through “acquisition of accreditation” (2022), and into the “early post-accreditation maintenance phase” (2023–2024). This allows for the analysis of QMS dynamics during its most unstable and formative period, which is the core focus of this study.

Rationale for the Number of Assessments (4 internal, 3 external): The included assessments represent all complete, formal audit cycles available within this defined study phase. The four internal audits correspond to the annual audits conducted from 2021 to 2024. The three external assessments correspond to the full external review cycle required for accreditation: the pre-accreditation assessment (2022), the surveillance review (2023), and the first reassessment (2024). This ensures the comprehensiveness and longitudinal comparability of the dataset.

We wish this addition adequately clarifies the fundamental rationale of the study design and makes the Methods section more rigorous and transparent.

The use of Sankey diagrams is innovative for visualizing flow relationships. However, the methodology for constructing these diagrams is not described in detail.

Thank you for acknowledging the innovativeness of our use of Sankey diagrams and for the important suggestion to provide a detailed methodology.

Accordingly, we have added a detailed description of the Sankey diagram construction process in the ***“Data Analysis” of Methods section (page3).***

The key points of the added description are as follows:

1.Node Definition: We detailed the definition of source and target nodes: in this study, the source nodes were the categories of NCs and root causes, and the target nodes were the categories of root causes and corrective actions, respectively.

2.Quantification and Linkage: We described the key steps in the construction process, including: (a) quantifying the frequency of occurrences from each source node to its respective target node; and (b) establishing links between the source and target nodes based on these frequency data (link width is proportional to frequency).

3.Visualization Purpose: We reiterated that the diagrams aim to visually illustrate the quantitative relationships between the source and target nodes.

We wish this added detail adequately fulfills your request, providing readers with a clear and reproducible workflow for constructing the diagrams.

The terms “recurrence rate and corrective action effectiveness” are provided, but the formulas could be more clearly formatted for readability.

Thank you for highlighting the importance of formula readability.

In response, we confirm that all formulas and their detailed computational methods are already provided in full in the ***“Data Analysis” section (page3)***. This includes:1.NC Recurrence Rate，2.Annual Cumulative Corrective Action Effectiveness Rate

3.Corrective Action Effectiveness Rate.Each formula is accompanied by clear variable definitions and computational explanations. In the revised manuscript, we have further enhanced the structure and readability through dedicated paragraphs, numbered listing, and clear variable notation.

We wish this provides sufficient methodological detail for reproducibility and hope the current formatting addresses your concern regarding readability.

In the result sections, table 1 is dense and could benefit from clearer subheadings or colour coding to distinguish internal VS. external data.

Thank you for the valuable suggestion regarding the readability of the tables.

We have undertaken a systematic optimization and restructuring of the tables ***in the Results section(page4).*** The specific improvements are as follows:1.Table Splitting and Hierarchical Enhancement: We have divided the original, densely populated Table 1 into three separate tables (NC distribution, root cause , and corrective action ). Each resulting table is now accompanied by a descriptive subheading, creating a clearer information hierarchy and a more focused presentation.2.Visual Distinction via Formatting: Across all tables comparing internal and external assessments, we have consistently applied bold italics to highlight data from external assessments. This provides an intuitive and uniform visual coding, allowing readers to quickly identify and contrast data from the two assessment sources.

We wish these improvements—splitting the table, adding subheadings, and employing bold italics for visual distinction—collectively and effectively address your suggestion for "clearer subheadings or colour coding to distinguish internal vs. external data."

Effect size or confidence intervals are missing, which limit interpretation of practical relevance.

Thank you for this important methodological suggestion. We have made the following substantial additions to the manuscript to ensure complete and interpretable statistical reporting:

Pre-specification in the Methods Section***（Page3）***: We have explicitly added to the statistical analysis description that effect sizes along with their 95% confidence intervals will be systematically reported.

Complete Presentation in Results Tables***（Page6-9）***: All relevant statistical comparison tables have been updated to directly include the calculated effect size and their 95% confidence intervals, providing readers with transparent and complete quantitative evidence.

We wish that by systematically incorporating this information across the Methods, Results sections, we have fully addressed your concern. This significantly enhances the statistical rigor, transparency of results, and practical guidance value of our study. We thank you for your valuable feedback.

Table 4 is insightful but underexplored. More discussion on why certain NCs recur despite corrective actions strengthen the findings.

Thank you for this insightful comment. In active response to your suggestion, we have added a dedicated, in-depth analysis  to ***Paragraph 5 of the Discussion section (Page 11-12)***. This analysis, based on a meticulous review of the data, systematically identifies four key systemic reasons for recurrence and proposes concrete improvement strategies:1.Insufficient Scope of Corrective Action Implementation.2.Superficial and Biased Review of Similar Issues.3.Inadequate Verification of Training Effectiveness.4.Cross-Departmental Coordination Gaps.

We wish this added discussion not only directly addresses your request for a "more in-depth exploration" of Table 4 but also advances the study's contribution from "describing phenomena" to  "providing structured solutions" . We thank you again for your guidance.

Although, authors acknowledge its single-centre limitation, which is commendable. However, it could propose specific strategies for multicentre validation.

We fully agree that proposing specific strategies for multicenter validation is crucial .We have made additions to the ***last Paragraph of the Discussion section (Page 12)***. We list “conducting a multicenter validation study” as a core future endeavor and systematically proposing the following strategies:1.Systematic Collection of Multi-Dimensional, Multi-Regional Laboratory Quality Data.2.Leveraging Artificial Intelligence to Identify and Screen Key Quality Indicators.3.Establishing an Automated, Intelligent Quality Assessment System as a Validation Platform. We thank you again for your guidance, which has helped us significantly enhance the rigor and foresight of our study.

The ethical approval or data protection is missing, which are important even in retrospective analyses.

In response to your comment, we have added a dedicated statement  in the “Data collection”subsection of the Methods ***(Page2).*** Administrative Approval: We emphasize that the data collection were formally approved by the laboratory administration.We thank you again for your review.

Checklist templates and training modules are needed in addition to the practical recommendation.

We have added explicit content to ***Paragraph 5 of the Discussion section (Page 11)***, providing clear development directions and design principles for two key tools: “training modules” and “checklist templates ”.

Framework for Training Modules:1.Format: Adopt face-to-face, repeated training sessions.2.Trainer Competency: Ensure trainers possess strong system knowledge and communication skills.3.Assessment: Conduct assessments on a day separate from training to ensure validity.4.Content: Focus on high-recurrence root causes, such as “root cause analysis” and “corrective action verification.”

Design Guidelines for Checklist TemplatesWe explicitly recommend: “Checklist templates should be designed to correspond directly to ISO 15189 requirements, allowing for sequential verification of each clause.
