# Supplementary material for: The ‘Double Helix’ model of quality monitoring: Risk mapping of quality management system during initial ISO 15189 Implementation in a medical laboratory
Source: PLoS One. 2026 Feb 4;21(2):e0342129. doi: 10.1371/journal.pone.0342129 (PMC12871953; doi:10.1371/journal.pone.0342129)
Supplement: Attachment — Submitted filename: Response to Reviewers-2.doc [file pone.0342129.s008.doc]

Dear Editor,

Thank you very much for taking the time to review our manuscript and providing us with timely feedback. We have carefully addressed all the comments and suggestions in the revised version.

We sincerely appreciate your guidance and support, which have enabled us to receive valuable feedback from you and the reviewers so promptly. Should any part of our revisions still fall short of your expectations, possibly due to our occasional misinterpretation of certain points, we kindly ask for your understanding, and we will continue to improve the manuscript accordingly.

Once again, we extend our heartfelt gratitude to you and the reviewers for your careful and constructive comments. The process of revising this manuscript has been a valuable opportunity for us to enhance our academic skills.

As the new year begins, we wish you a happy, healthy, and successful year.

Sincerely

Journal Requirements:

If the reviewer comments include a recommendation to cite specific previously published works, please review and evaluate these publications to determine whether they are relevant and should be cited. There is no requirement to cite these works unless the editor has indicated otherwise.

Thank you for your reminder. We have carefully reviewed all the reviewer comments and would like to clarify that they did not include specific recommendations to cite additional previously published works.

Please review your reference list to ensure that it is complete and correct. If you have cited papers that have been retracted, please include the rationale for doing so in the manuscript text, or remove these references and replace them with relevant current references. Any changes to the reference list should be mentioned in the rebuttal letter that accompanies your revised manuscript. If you need to cite a retracted article, indicate the article’s retracted status in the References list and also include a citation and full reference for the retraction notice.

Thank you for your guidance regarding the reference list. In response to your comments, we have taken the following actions:1.We have carefully reviewed our entire reference list to ensure its completeness and accuracy.2.Each reference has been verified against the PubMed database, and none of the cited papers have been retracted.3.The formatting of all references has been updated to comply with the journal's specific style guide.

These updates have been made in the revised manuscript. Thank you again for your valuable oversight.

Reviewer comments:

Recommendation

Accept after minor editorial revision.The manuscript now meets the standards of rigour, transparency, and contribution expected for publication, offering a meaningful and practically relevant addition to the literature on ISO 15189 implementation and quality management in medical laboratories.

Thank you for your guidance.In accordance with the journal's formatting guidelines, we have made the following editorial adjustments to the manuscript:1.****Paragraph Alignment****: The left margin has been unified throughout the main text. For ****Table 4**** and ****Table 5****, due to their substantial content and necessary internal formatting to ensure clarity, the paragraph indentation within the tables differs slightly from the main body text. ****2.Paragraph Formatting****: We have ensured that ****no paragraph begins with an indentation****, and a clear blank line now separates each paragraph.

We believe these formatting updates fully align with the journal's style requirements. Thank you once again for your and the reviewers' support throughout the review process.
