# Supplementary material for: The ‘Double Helix’ model of quality monitoring: Risk mapping of quality management system during initial ISO 15189 Implementation in a medical laboratory
Source: PLoS One. 2026 Feb 4;21(2):e0342129. doi: 10.1371/journal.pone.0342129 (PMC12871953; doi:10.1371/journal.pone.0342129)
Supplement: Attachment — Submitted filename: plosfinal rev helix.docx [file pone.0342129.s009.docx]

It is verified that the authors have completely complied with the necessary revisions after examining the reviewer/editor comments, the authors' reply letter, and the amended manuscript. The reference list has been carefully examined for accuracy and completeness, checked against PubMed to make sure no works that have been retracted are cited, and structured in compliance with the journal's style rules. Furthermore, all minor editing and formatting changes that were requested—such as paragraph alignment, spacing, and adherence to journal formatting guidelines—have been suitably applied. There are no unresolved concerns pertaining to the reviewer's remarks, and the authors' responses appropriately reflect the modifications made in the revised article.
