# Supplementary material for: The ‘Double Helix’ model of quality monitoring: Risk mapping of quality management system during initial ISO 15189 Implementation in a medical laboratory
Source: PLoS One. 2026 Feb 4;21(2):e0342129. doi: 10.1371/journal.pone.0342129 (PMC12871953; doi:10.1371/journal.pone.0342129)
Supplement: Attachment — Submitted filename: Reviewer comments-Helix.docx [file pone.0342129.s007.docx]

The authors have thoroughly and conscientiously revised the manuscript in response to the reviewers’ comments. The revised version demonstrates substantial improvement in clarity, methodological rigour, conceptual coherence, and scholarly depth, and the previously identified major weaknesses have been adequately addressed.

**Notably, the authors have strengthened the manuscript by:**

Clearly defining and operationalising the "Double Helix" model, grounding it in empirical findings and illustrating it with a well-integrated schematic. The model is no longer merely metaphorical but emerges logically from the interaction between internal and external assessments.

Significantly enhancing methodological transparency, including explicit inclusion criteria, detailed definitions and formulas for recurrence and corrective action effectiveness rates, and the introduction of a robust dual-review and adjudication process to ensure data reliability.

Improving statistical reporting and data presentation, with appropriate justification of statistical tests, inclusion of effect sizes and confidence intervals, restructured tables, and well-explained figures (including Sankey diagrams).

Deepening the Discussion, moving beyond descriptive reporting to provide analytical insight into the causes of NC recurrence, the differential roles of internal and external assessments across stages of QMS development, and practical, actionable frameworks for laboratories implementing ISO 15189.

Appropriately acknowledging limitations, including single-centre design, assessor variability, and sample size constraints, while also proposing realistic and forward-looking strategies for multicenter validation and digital/AI-supported quality monitoring.

Ensuring compliance with journal requirements, including ethics, data availability, and reporting standards.

While a small number of minor editorial and language refinements could further

enhance the polish (e.g., occasional repetition or typographical errors), these issues do not detract from the study's scientific validity, originality, or practical relevance and can be readily addressed during copyediting.

Recommendation

Accept after minor editorial revision. The manuscript now meets the standards of rigour, transparency, and contribution expected for publication, offering a meaningful and practically relevant addition to the literature on ISO 15189 implementation and quality management in medical laboratories.
